# Cathelicidin-BF, a Snake Cathelicidin-Derived Antimicrobial Peptide, Could Be an Excellent Therapeutic Agent for Acne Vulgaris

**DOI:** 10.1371/journal.pone.0022120

**Published:** 2011-07-15

**Authors:** Yipeng Wang, Zhiye Zhang, Lingling Chen, Huijuan Guang, Zheng Li, Hailong Yang, Jianxu Li, Dewen You, Haining Yu, Ren Lai

**Affiliations:** 1 Biotoxin Units of Key Laboratory of Animal Models and Human Disease Mechanisms, Kunming Institute of Zoology, Chinese Academy of Sciences, Kunming, Yunnan, China; 2 College of Life Sciences, Hebei Normal University, Shijiazhuang, Hebei, China; 3 School of Life Science and Biotechnology, Dalian University of Technology, Dalian, Liaoning, China; 4 Clinical Laboratory, The First Affiliated Hospital of Kunming Medical College, Kunming, Yunnan, China; 5 Graduate School of the Chinese Academy of Sciences, Beijing, China; University of Leuven-Rega Institute, Belgium

## Abstract

Cathelicidins are a family of antimicrobial peptides acting as multifunctional effector molecules in innate immunity. Cathelicidin-BF has been purified from the snake venoms of *Bungarus fasciatus* and it is the first identified cathelicidin antimicrobial peptide in reptiles. In this study, cathelicidin-BF was found exerting strong antibacterial activities against *Propionibacterium acnes*. Its minimal inhibitory concentration against two strains of *P. acnes* was 4.7 µg/ml. Cathelicidin-BF also effectively killed other microorganisms including *Staphylococcus epidermidis*, which was possible pathogen for acne vulgaris. Cathelicidin-BF significantly inhibited pro-inflammatory factors secretion in human monocytic cells and *P. acnes*-induced O_2_^.−^ production of human HaCaT keratinocyte cells. Observed by scanning electron microscopy, the surfaces of the treated pathogens underwent obvious morphological changes compared with the untreated controls, suggesting that this antimicrobial peptide exerts its action by disrupting membranes of microorganisms. The efficacy of cathelicidin-BF gel topical administering was evaluated in experimental mice skin colonization model. *In vivo* anti-inflammatory effects of cathelicidin-BF were confirmed by relieving *P. acnes*-induced mice ear swelling and granulomatous inflammation. The anti-inflammatory effects combined with potent antimicrobial activities and O_2_^.−^ production inhibition activities of cathelicidin-BF indicate its potential as a novel therapeutic option for acne vulgaris.

## Introduction

Antimicrobial peptides play important roles in preventing microorganism infections. Most of them are 10–50 residues in length. They can provide an effective and fast acting defense against harmful microorganisms [1], [2]. There are two major vertebrate antimicrobial peptide families including cathelicidins and defensins. Cathelicidins have been found in many mammalians and birds. Recently, a few cathelicidin antimicrobial peptides were identified from snake venoms [3], [4]. They are the first report of reptile cathelicidins.

Acne vulgaris is the most common skin disease. It often occured in areas containing large skin oil glands, such as face, back, and trunk [5]. The pathogenesis of acne is currently attributed to multiple factors such as hormonal factors, hyperkeratinization, resident microbiota, sebum, nutrition, cytokines and toll-like receptors [6]. *Propionibacterium acnes* act on important roles in acne pathogenesis although they belong to the resident microbiota. *P. acnes* induce the expression of antimicrobial peptides and pro-inflammatory cytokines/chemokines, which contribute to the inflammatory responses of acne [7]–[10]. Some *P. acnes* strains may cause an opportunistic infection worsening acne lesions [6]. Antibiotics are employed as therapeutic agents for acne by inhibiting inflammation or killing bacteria. However, antibiotic resistance has been increasing in prevalence within the dermatologic setting [11]. Antimicrobial peptides have been considered as new type of antimicrobial reagents because they have low potential to induce drug resistance of microorganisms. The current work is performed to evaluate the anti-*P. acnes* abilities of cathelicidin-BF *in vitro* and *in vivo*.

## Results

### Antimicrobial activities of cathelicidin-BF

As listed in Table 1, cathelicidin-BF showed strong antimicrobial abilities against several microorganisms, which are related to acne vulgaris. The MICs value of cathelicidin-BF, LL-37 and clindamycin against two *P. acnes* strains are 4.7 µg/ml (1.3 µM), 9.4 µg/ml (2.2 µM), and 2.3 µg/ml (5.2 µM), respectively. Cathelicidin-BF showed strong antimicrobial functions (MIC of 1.2–2.3 µg/ml, 0.33–0.65 µM) against two strains of *S. epidermidis* while LL-37 has no activity against them (no amtimicrobial activity was seen when the concentration of LL-37 was up to 200 µg/ml, 46.8 µM). Clindamycin showed only antimicrobial ability (MIC of 1.2 µg/ml, 2.6 µM) against one (*S. epidermidis*09B2490) of the two *S. epidermidis* strains. The MIC values of cathelicidin-BF and LL-37 against *S. aureus* ATCC2592 are 4.7 µg/ml (1.3 µM for cathelicidin-BF, 1.1 µM for LL-37) while that of clindamycin is 1.2 µg/ml (2.6 µM).

**Table 1:** Antimicrobial activities of cathelicidin-BF.

	MIC (µg/ml)		
Microorganisms	BF	LL-37	CL
*P. acnes* ATCC6919	4.7 (1.3 µM)	9.4 (2.2 µM)	2.3 (5.2 µM)
*P. acnes* ATCC11827	4.7 (1.3 µM)	9.4 (2.2 µM)	2.3 (5.2 µM)
*S. epidermidis* 09A3726	2.3 (0.65 µM)	NA	NA
*S. epidermidis* 09B2490	1.2 (0.33 µM)	NA	1.2 (2.6 µM)
*S. aureus* ATCC2592	4.7 (1.3 µM)	4.7 (1.1 µM)	1.2 (2.6 µM)

MIC: minimal peptide concentration required for total inhibition of cell growth in liquid medium. These concentrations represent mean values of three independent experiments performed in duplicates.

BF: canthelicidin-BF; CL: clindamycin.

### Bacteria killing kinetics

**Figure 1:**
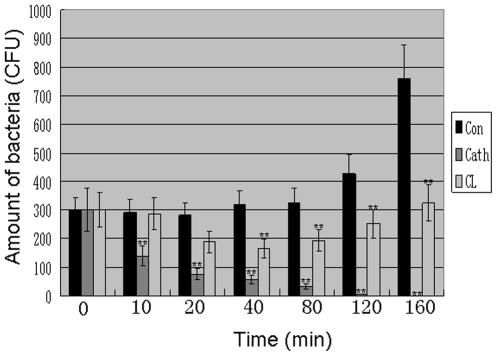
Bacterial killing kinetics of cathelicidin-BF against *P. acnes*. Amount of bacteria co-cultured with different sample for different time (CFU) was counted according to method described in the “Materials and Methods” section. These CFU represent mean values of three independent experiments. The values for cathelicidin-BF and clindamycin were significant different from the values for the control (*P<0.05 and **p<0.01). Con: control; Cath: cathelicidin-BF; CL: clindamycin.

Using clindamycin as a positive control, antibacterial properties of cathelicidin-BF against *P. acnes* ATCC6919 were tested by the colony counting assay. As illustrated in Fig. 1, cathelicidin-BF could rapidly exert its antibacterial activities. It just took less than 160 minutes to kill all the *P. acnes* at the concentration of one time of MIC. The antibacterial activity was proved to be lethal for *P. acnes*. *P. acnes* were not capable of resuming growth on agar plates after a 6-h treatment with concentrations above the corresponding MICs. In contrast, the antibiotics, clindamycin could not clean the bacteria at the concentration of one time of MIC. Besides, *P. acnes* treated by one time MIC of clindamycin was capable of resuming growth after 80 min of the treatment (Fig. 1).

### The Effects on membrane morphology

**Figure 2:**
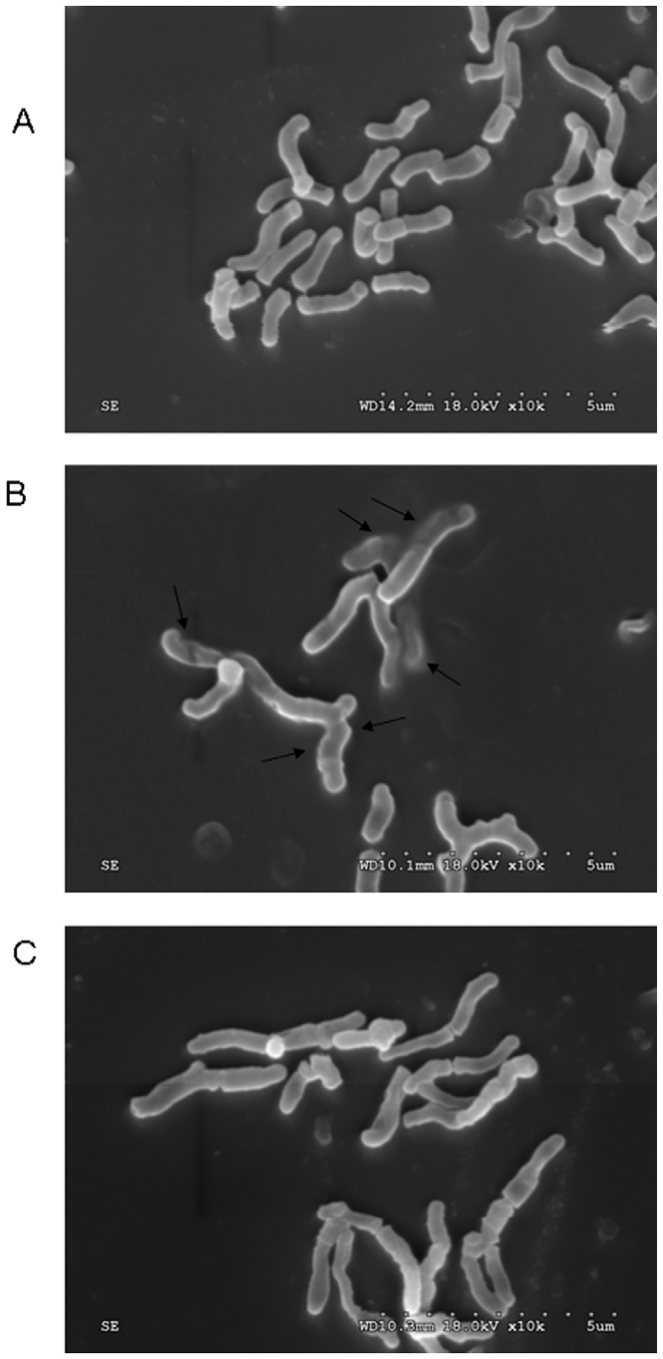
Scanning electron micrographs of control (A), cathelicidin-BF-treated (B), and clindamycin-treated (C) *P. acnes*. The arrows indicate damage to the plasma membranes of bacteria or the intracellular inclusions efflux.

The morphology difference of untreated, clindamycin-treated and cathelicidin-BF-treated *P. acnes* was studied by SEM as illustrated in Fig. 2. There are clear morphology differences among these *P. acnes*. The outer membranes of untreated *P. acnes* were long, spindle-shaped, and smooth (Figs. 2A). But once treated with cathelicidin-BF, the intracellular inclusions were found effluxed extracellularly (the representatives are indicated by arrows), indicating that the breaks might be formed in the plasma membranes of *P. acnes*. In addition, the cell swell was observed obviously in the cathelicidin-treated *P. acnes* (Fig. 2B). Clindamycin-treated *P. acnes* had no significant morphology difference from the untreated bacterium (Fig. 2C), suggesting that it does not act on membranes. In fact, clindamycin kills bacteria by inhibiting protein synthesis.

### Cathelicidin-BF inhibits P. acnes-induced O_2_^.−^ production

**Figure 3:**
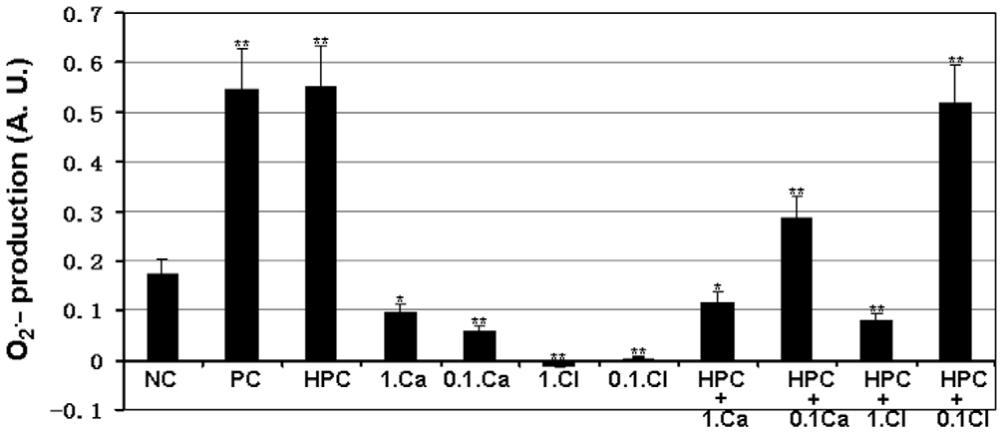
Effects of cathelicidin-BF and clindamycin on ROS production by *P. acnes*-stimulated keratinocytes. The human HaCaT keratinocyte cells were incubated with 1×10^6^ bacteria at in the presence of test sample 37°C for 18 h. These represent mean values of three independent experiments. The values for cathelicidin-BF and clindamycin were significant different from the value for the negative control (*P<0.05 and **p<0.01). NC: Negative control; PC: live *P. acnes*; HPC: heat-killed *P. acnes*; Ca: cathelicidin-BF; CL: clindamycin; 1. and 0.1.:, 1× MIC and 0.1× MIC.

Previous work by Grange et al has indicated that *P. acnes* significantly induces O_2_^.−^ production, which affects IL-8 levels [12]. The effects of cathelicidin-BF on *P. acnes*-induced O_2_^.−^ production was tested in this work. Both live and heat-killed *P. acnes* significantly induced O_2_^.−^ production as the results from Grange et al [16]. As illustrated in Fig. 3, cathelicidin-BF with 1× or 0.1× MIC could significantly inhibited *P. acnes*-induced O_2_^.−^ production. Different from cathelicidin-BF, only 1× MIC clindamycin significantly inhibited *P. acnes*-induced O_2_^.−^ production but 0.1× MIC clindamycin had little effect.

### Cytokine production inhibition by cathelicidin-BF

**Figure 4:**
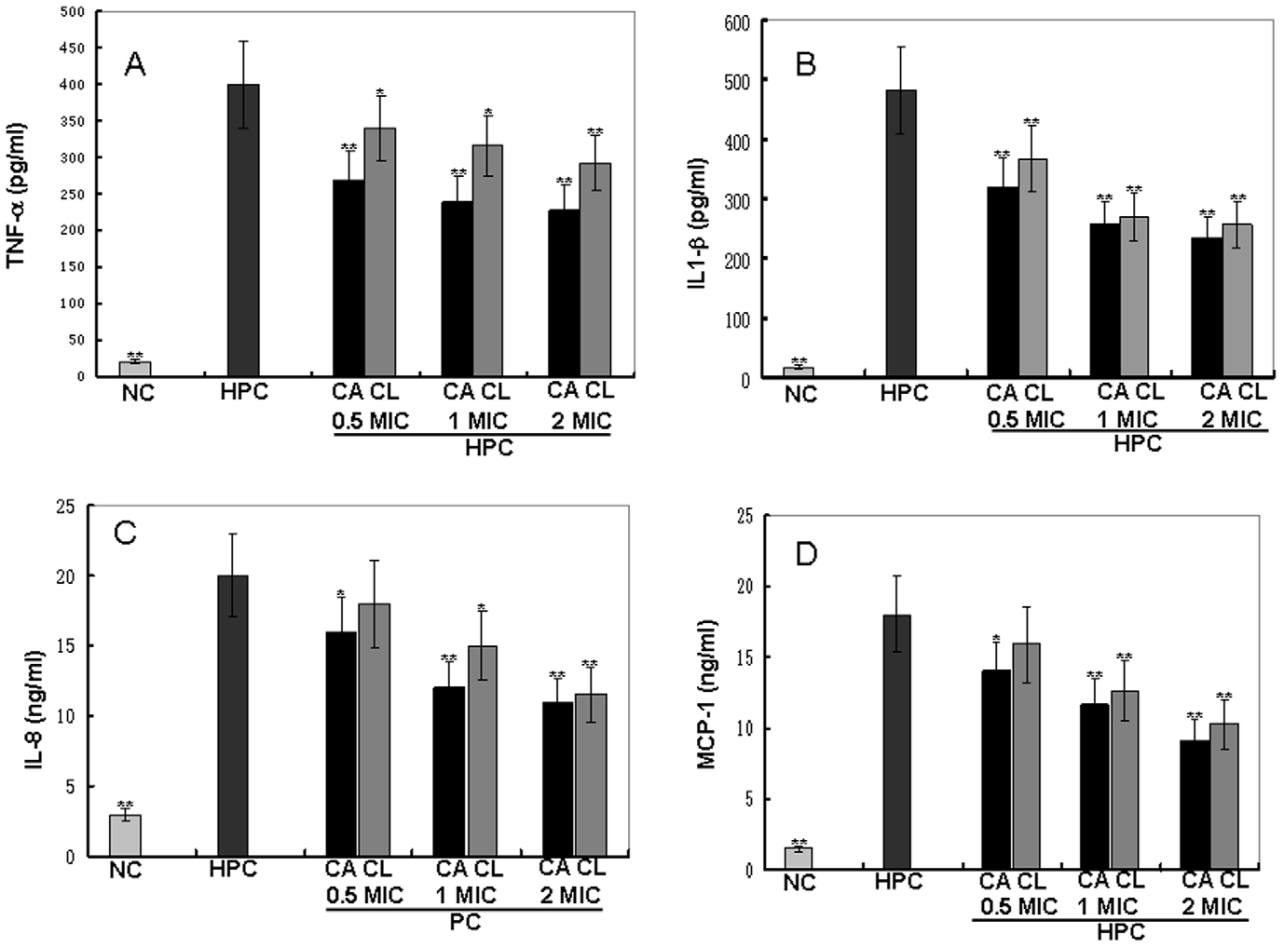
Effects of cathelicidin-BF and clindamycin on cytokine production by *P. acnes*-stimulated monocytic cells. Human monocytic THP-1 cells (1×10^6^ cells/ml) were incubated with heat-killed (incubated at 80°C for 30 min to kill the bacteria) *P. acnes* (wet weight 100 µg/ml) alone or in combination with different concentrations (0.01, 0.05, and 0.1 mg/ml) of tested sample for 18 h. These represent mean values of three independent experiments. The values for cathelicidin-BF and clindamycin were significant different from the value for the HPC group (*P<0.05 and **p<0.01). NC: Negative control; HPC: heat-killed *P. acnes*; CA: cathelicidin-BF; CL: clindamycin.

Several pro-inflammatory cytokines including TNF-α, IL-1β, IL-8, and MCP-1 were induced by heat-killed *P. acnes* as listed in Fig. 4. Both cathelicidin-BF and clindamycin could significantly inhibit cytokines' secretion induced by *P. acnes* in a dose-dependent manner. For example, TNF-α, one of the most important pro-inflammatory cytokines was induced to a concentration of 400 pg/ml by the heat-killed *P. acnes* (Fig. 4A). 0.5, 1, and 2 times of MIC of cathelicidin-BF could inhibit 32.5, 40.3, and 43.3% of the induced TNF-α secretion, respectively, while the inhibition rate of clindamycin was 14.7, 20.7, and 27%, respectively. To account for any reduction in pro-inflammatory cytokines resulting from cytotoxic effects of cathelicidin-BF, the cytotoxicity induced by these extracts was determined by MTT assays in THP-1 cells. cathelicidin-BF had little cytotoxic effects with only 0.3, 0.7, and 1.4% cell growth inhibition at concentration of 0.5, 1, and 2 times of MIC, respectively. In addition, after an 18-h incubation, only cathelicidin-BF (no heat-killed *P. acnes*) did not increase the secretions of either TNF-a, IL-8, IL-1b, or MCP-1 by THP-1 cells.

### *In vivo* mice ear colonization inhibition of *P. acnes* and anti-inflammation by cathelicidin-BF

**Figure 5:**
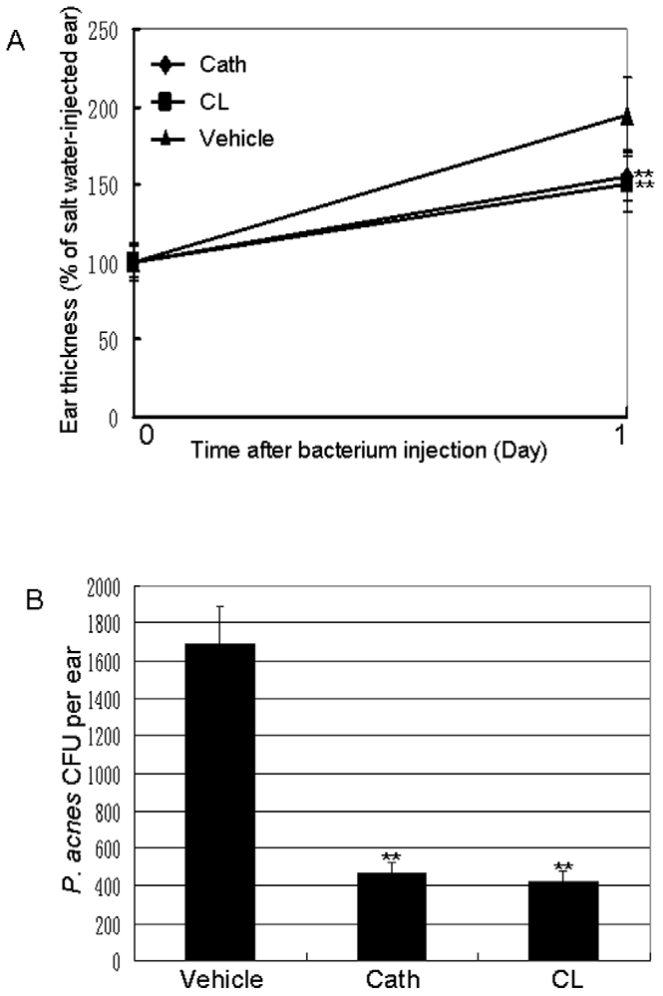
Effects of 0.2% cathelicidin-BF and clindamycin gel on *P. acnes*-induced inflammation and *P. acnes* growth *in vivo*. Left ears of mice were intradermally injected with *P. acnes* (1×10^7^ CFU per 20 µl in PBS) to induce inflammation. Right ears of the same mice were injected with 20 µl of 0.9% salt water (vehicle). Subsequently, 0.2% cathelicidin-BF gel, 0.2% clindamycin gel or vehicle was applied on the ear skin surface of mice. (**A**) The increase in ear thickness was measured using a micro caliper before and 24 hours after the bacterial injection. (**B**) 24 hours after P. acnes injection, CFUs of P. acnes in the ear were enumerated as described in the Materials and methods” section. Data represent mean ± SE of five individual experiments. Cath: cathelicidin-BF; CL: clindamycin. The values for cathelicidin-BF and clindamycin were significant different from the value for the vehicle (*P<0.05 and **p<0.01).

Intradermally injected *P. acnes* induced severe inflammation in the ears of Kunming mice as illustrated in Fig. 5A. One day after the injection, the ear thickness was about two time of the control. Both cathelicindin-BF and clindamycin 0.2% gels could inhibit the inflammation induced by *P. acnes*. After one day treatment, 0.2% cathelicindin-BF (425 times of MIC) and clindamycin (950 times of MIC) gel could inhibit 42% and 47% of the ear swelling, respectively.

The numbers of *P. acnes* colonized within the ears were illustrated in Fig. 5B. The number of *P. acnes* colonized within the ear treated by the vehicle is about 1700. The number of *P. acnes* in the cathelicindin-BF and clindamycin group is about 470 and 430, respectively. Furthermore, data from MTT assays showed that at concentration of 1, 10, and 30 times of MIC, cathelicindin-BF inhibited 0.2, 1.1, and 1.9% cell growth, respectively, indicating almost no cytotoxicity on human HaCaT keratinocyte cells. These data suggest that dermal application of cathelicindin-BF can effectively relieve *P. acnes*-induced inflammation without detrimental effects on skin cells.

## Discussion

Over the past years, natural antimicrobial peptides (AMPs) have attracted considerable interests as a new type of antimicrobial agents for several reasons including their relative selectivity towards targets (microbial membranes), their rapid mechanism of action and, above all, the low frequency in selecting resistant strains [13]–[15]. Cathelicidin-BF is an antimicrobial peptide identified from the snake venoms of *B. fasciatus*. Our previous work has indicated that cathelicidin-BF exerted strong and rapid antimicrobial activities against many microorganisms including Gram-negative, Gram-positive bacteria and fungi, especially some clinically isolated drug-resistance microorganisms. Besides, cathelicidin-BF has no hemolytic and cytotoxic activity on human cells [3]. However, its effect toward *P. acnes* has not been studied.

Several antimicrobial peptides including epinecidin- and granulysin-derived peptides, and frog skin peptides have been found to exert anti-*P. acnes* functions [16]–[18]. Recently, sebocytes are found to express functional cathelicidin antimicrobial peptides with activity to kill *P. acnes* [12]. Considering anti-inflammatory activities of some antimicrobial peptides, they are suggested to be potent agents for acne vulgaris treatment [13]. The current work indicated that cathelicidin-BF contained potential antimicrobial activity against *P. acnes in vitro* (Table 1, Fig. 1). Its MIC against two *P. acnes* strains is 4.7 µg/ml (1.3 µM), which is comparable to the anti-*P. acnes* potential antibiotics of clindamycin (2.3 µg/ml, 5.2 µM) (Table 1). SEM study indicated that cathelicidin-BF acted on the membrane of *P. acnes* (Fig. 2).

Previous work by Grange *et al* has indicated that the whole *P. acnes* bacteria or the extract of its surface proteins had the same effects to induce O_2_^.−^ production in keratinocytes. In addition, their results also indicted that the toxicity of reactive oxygen species on *P. acnes*-stimulated keratinocytes is mainly caused by the O_2_^.−^ overproduction [12]. In this study, cathelicidin-BF was found to obviously inhibit O_2_^.−^ production induced by *P. acnes* in the HaCaT keratinocyte cells (Fig. 3). By O_2_^.−^ production inhibition, cathelicidin-BF may inhibit inflammation because O_2_^.−^ can exert a positive effect on IL-8 production [12].

Some factors of *P. acnes*, such as heat shock protein HSP60, can stimulate the production of pro-inflammatory cytokines IL-1b and TNF-a [19]. In turn, these released cytokines lead to the inflammatory reactions [20]. The anti-inflammatory function of cathelicidin-BF was evaluated by measuring its effects on pro-inflammatory cytokine secretion. It could significantly inhibit *P. acnes*-induced secretion of several pro-inflammatory factors including TNF-a, IL-8, IL-1b, and MCP-1 *In vitro* (Fig. 4). *In vivo* anti-inflammatory effect of cathelicidin-BF was confirmed by relieving *P. acnes*-induced ear swelling and granulomatous inflammation (Fig. 5). The anti-inflammatory effects combined with potent antimicrobial activities and O_2_^.−^ production inhibition activities of cathelicidin-BF indicate its pontential as a novel therapeutic option for acne vulgaris.

## Materials and Methods

### Peptides synthesis

Two cathelicidins (snake cathelicidin-BF, KFFRKLKKSVKKRAKEFFKKPRVIGVSIPF, and human cathelicidin, LL-37, LLGDFFRKSKEKIGKEFKRIVQRIKDFLRNLVPRTES) were synthesized by GL Biochem (Shanghai) Ltd. (Shanghai, China) and analyzed by HPLC and mass spectrometry to confirm their purity higher than 98%.

### Microorganism strains and growth conditions

*Propionibacterium acnes* (ATCC6919 and ATCC 11827), *Staphylococcus epidermidis* (09A3726 and 09B2490) and *Staphylococcus aureus* (ATCC 2592) were obtained from Kunming Medical College. *P. acnes* were cultured in brain heart infusion (BHI) broth (HKM,Guangzhou, China) with 1% glucose at 37°C for 3 days to exponential-phase and for 5 days to stationary phase. The bacteria were cultured in an anaerobic atmosphere using MGC Anaeropack systems (Mitsubishi Gas Chemical Co., Inc, Japan); *S. epidermidis* and *S. aureus* were grown in LB (Luria-Bertani) broth as our previous report [21].

### Susceptibility testing

MIC (minimal inhibitory concentration) of antimicrobial peptides against microorganisms was determined using broth dilution determination as our previous methods [22]. Peptides were prepared as a stock solution in H_2_O at a series of concentration. 890 µl special broth (BHI broth for *P. acnes*, LB broth for *S epidermidis* and *S. aureus*), 100 µl bacterial suspension (10^8^ CFU/ml) and 10 µl test peptides were put together in the test tube and shaken at 37°C for 24 h. A tube with corresponding volume of H_2_O was used as control. The MIC was defined as the lowest concentration of test peptides inhibiting microorganism's growth.

### Bacteria killing kinetics

The bacterial effect of cathelicidin-BF against *P. acnes* (ATCC6919) was tested using clindamycin as positive control. *P. acnes* was grown to log phase in BHI broth and centrifuged at 5000 rpm for 5 min. The collected bacterium pellet was washed twice by BHI broth and diluted to 1×10^6^ CFU/ml with BHI broth. Cathelicidin-BF or clindamycin with one time of MIC was added into the BHI broth containing *P. acnes* and cultured at 37°C with shaking. The colony counting was performed at different times as described by Mygind *et al* [23].

### Scanning electron microscopy (SEM)

SEM was performed to study the possible mechanisms of action of cathelicidin-BF on bacteria according to the methods described by Lu *et al* [15] with minor modification. *Propionibacterium acnes* ATCC6919 was cultured in BHI liquid medium to exponential-phase. After washing with 0.15 M sodium chloride solution for two times, the bacteria were resuspended and incubated with cathelicidin-BF (1× MIC) at 37°C for 30 min. The pellets after centrifuging at 1000 rpm for 10 min were fixed with 2.5% buffered glutaraldehyde at 4°C for 2 h. The bacteria were then postfixed in 1% buffered osmium tetroxide for 2 h, dehydrated in a graded series of ethanol, frozen in liquid nitrogen cooled tertbutyl alcohol and vacuum dried overnight. After mounting onto aluminum stubs and vacuum sputter-coating with gold, the samples were analyzed with a Hitachi S-3000N SEM under standard operating conditions.

### Measurement of O_2_^.−^ production

Measurement of O_2_^.−^ production was carried out following the protocol described by Grange *et al* [24]. The human HaCaT keratinocyte cells (1×10^5^ cells/ml, obtained from Cell Bank of Kunming Institute of Zoology, Chinese Academy of Sciences) were cultured in 96-well plates with Dulbecco's modified Eagle's medium (DMEM, Gibco) containing 10% fetal calf serum and penicillin (100 u/ml)–streptomycin (100 µg/ml) at 37°C in a humidified 5% CO2 atmosphere. The cell line was routinely tested to assess the absence of *Mycoplasma* infection. The monolayer cells were washed three times with PBS (1.5 mM KH_2_PO_4_, 2.7 mM Na_2_HPO_4_.7H_2_O, 0.15 M NaCl, pH 7.4), and cultured with serum-free and antibiotic-free DMEM medium, incubating with heat-killed *P. acnes* (1×10^6^) in the presence of test samples. After co-culturing for 18 h, the cells were washed with PBS and incubated with 100 µl per wells of 5 µM dihydroethidium solution (DHE, a fluorescent superoxide anion indicator) for 30 min. The level of intracellular O_2_^.−^ was assessed by spectrofluorimetry (excitation/emission maxima: 480/610 nm) on a spectrofluorimeter (FlexStation 3, Molecular Devices, USA).

### Measurement of cytokine production in human monocytic cells

Human monocytic THP-1 cells (1×10^6^ cells/ml, Shanghai Caoyan Biotechnology Co. Ltd, China) were cultured in 24-well plates containing serum-free medium (RPMI 1640 medium, Gibco Life Technologies). They were added with heat-killed (incubated at 80°C for 30 min to kill the bacteria) *P. acnes* (wet weight 100 µg/ml) alone or in combination with different concentrations (0.01, 0.05, and 0.1 mg/ml) of tested samples for an 18-h incubation. Cell-free supernatants were collected, and concentrations of MCP-1, TNFa, IL-1b, and IL-8 were measured using corresponding enzyme immunoassay kits (Adlitteram Diagnostic Laboratories, Inc, USA).

### *In vitro* cytotoxicity of cathelicidin-BF on human skin and monocytic cells

THP-1 and HaCaT cells were cultured in 96-well plates as described above. Cell viability was evaluated by conventional 3-(4,5-dimethyl-2-thiazolyl)-2,5-diphenyl-2H-tetrazolium bromide (MTT) reduction assays. After a 24-h treatment by the test samples, 0.1 ml of MTT (5 mg/ml) was added to each well. The supernatant was removed after 2 h of incubation, and 100 µl acidic isopropanol was mixed with the precipitate. The absorbance at 540 nm of the resulting solution was measured. The experiments were performed in triplicate.

### *In vivo* mice ear colonization of *P. acnes*

*P. acnes* (ATCC6919) was grown to the exponential-phase in BHI broth. The bacterium was centrifuged at1000 rpm for 10 min. The bacterium pellet was washed twice with 0.15 M sodium chloride solution, and re-suspended in 0.15 M sodium chloride solution (5×10^8^ CFU/ml). *P. acnes* (1×10^7^ CFU per 20 µl) was intradermally injected into left ears of Kunming mice (20±2 g). Right ears received the same volume of 0.15 M sodium chloride solution. Placebo gel, cathelicidin-BF or clindamycin 0.2% gel (Polyethylene Glycol (PEG) 400∶ PEG 4000, 1∶1) were administered (nine mice per group) on the skin surfaces of ears and the sites were covered with OpSite dressings and occlusively sealed with adhesive tape. The increase of ear thickness after 24 h bacterial injection was measured using a micro caliper. The increase in ear thickness of the left ear was calculated as percentage of the right ear.

To determine *P. acnes* number in the ear, the left ear was cut off after 24 h bacterial injection and wiped to remove gel. The ear was homogenized in 0.15 M sodium chloride solution (1 ml per ear) withith a hand tissue grinder. CFUs of *P. acnes* in the ear were enumerated by plating serial dilutions of the homogenate on BHI plates. The plates were anaerobically incubated at 37°C for 72 hours and the bacterial numbers were counted. All the experimental protocols to use animals were approved by the Animal Care and Use Committee at Kunming Institute of Zoology, Chinese Academy of Sciences. The approval ID for this study was syxk2009-0026.

### Statistics

Data were analyzed by *X* ^2^ and by *t* test or repeated measure analysis of variance (ANOVA) comparison of means.
